# Multivariate Timing and Granger Causality Analysis of Spontaneous Facial Mimicry in Response to Android Dynamic Facial Expressions

**DOI:** 10.3390/s26061881

**Published:** 2026-03-17

**Authors:** Chun-Ting Hsu, Anna Kelbakh, Dongsheng Yang, Takashi Minato, Wataru Sato

**Affiliations:** 1 Psychological Process Research Team, Guardian Robot Project, RIKEN, Soraku-gun, Kyoto 619-0288, Japan; 2 Division of Psychology and Language Sciences, University College London, London WC1H 0AP, UK; 3 Graduate School of Informatics, Kyoto University, Yoshida-Honmachi, Sakyo, Kyoto 606-8501, Japan; 4 Interactive Robot Research Team, Guardian Robot Project, RIKEN, Soraku-gun, Kyoto 619-0288, Japan; takashi.minato@riken.jp

**Keywords:** human–android interaction, spontaneous facial mimicry, Facial Action Coding System, Py-feat, cross-correlation, vector autoregressive models

## Abstract

Although evidence exists for android-induced spontaneous facial mimicry, the timing and temporal precedence (Granger causality) of this effect remain uncertain. We used the Facial Action Coding System (FACS) to analyze simultaneous dyadic facial video recordings of participants observing android Nikola’s negative (frowning) and positive (smiling) dynamic facial expressions. Principal component analysis of Nikola’s expressions indicated that, in addition to the action units (AUs) 04 (brow lowerer) and 12 (lip-corner puller), AUs 25 (lips part) and 26 (jaw drop) contributed significantly to Nikola’s facial expressions. Cross-correlation analysis revealed AU04 mimicry of negative expressions and AU12 mimicry of positive expressions from 400 ms onwards. AU25 and AU26 mimicry occurred faster, starting at around 200 ms. Multilevel vector autoregression incorporated the android and participant AUs and quantified the temporal evolution of the Granger causality for the first time. In addition to paired android–human AU04, 12, 25, and 26 effects, significant Granger causality was found between different android–human AU combinations, such as from android AU04 to participant AU25 in the negative condition, and from android AU25 to participant AU12 in the positive condition. These results suggest that the spontaneous facial responses to Nikola’s expressions involved not only motor copying, but also higher-level goal emulation and motor planning in the mirror mechanism, supporting the reliability of the social function of android facial expressions. Cross-correlation and Granger causality analysis can be valuable when further investigating behavioral matching in real-life contexts.

## 1. Introduction

Spontaneous facial mimicry is a form of behavior matching [1] that commonly occurs during face observation and facilitates emotion contagion [2] and affective empathy [3,4]. Advances in robotics have enabled androids to produce human-like facial expressions [5,6]. Previous studies have demonstrated that humans can respond to an android’s facial expressions with spontaneous facial mimicry [6,7]. Classical approaches have relied on facial electromyography (EMG) to record muscle activation patterns that resemble presented facial stimuli [8,9,10,11,12]. Alternatively, facial action patterns of participants on video recordings have been manually or automatically coded to detect mimicry using the Facial Action Coding System (FACS) [13,14,15,16,17,18], which analyzes facial movements in terms of anatomically based action units (AUs) [19,20,21] and was designed for manual coding by human observers. Over the past decade, advances in computer vision and machine learning have enabled automated FACS coding.

**Figure 1:**
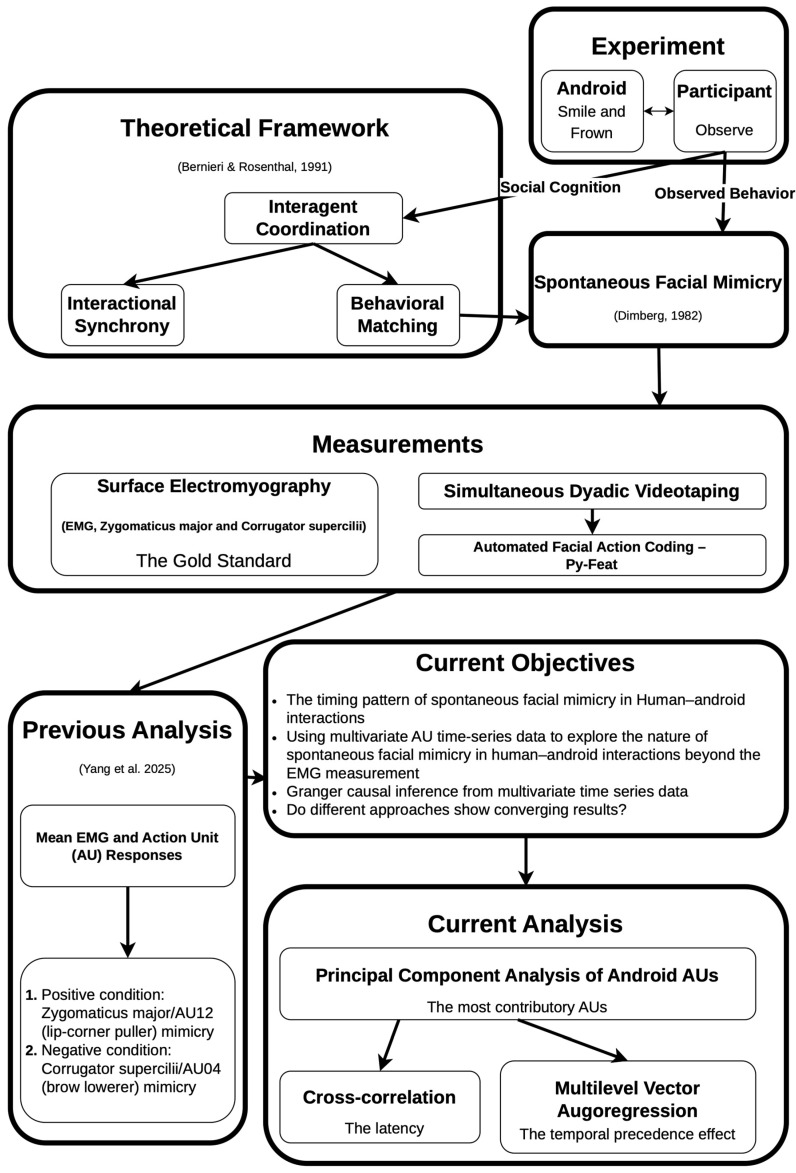
The theoretical and analytical frameworks of the present study [1,7,9].

**Figure 2:**
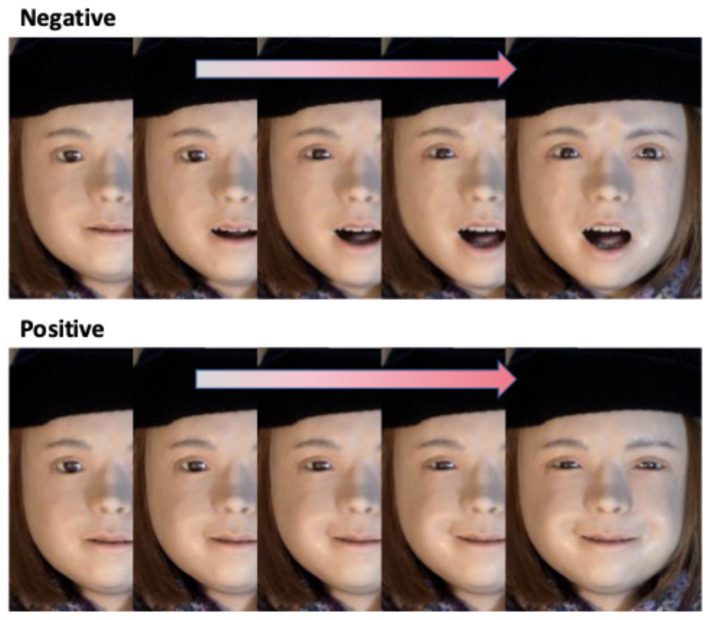
The android Nikola displaying dynamic facial expressions in the positive (smiling) and negative (frowning) conditions.

**Figure 3:**
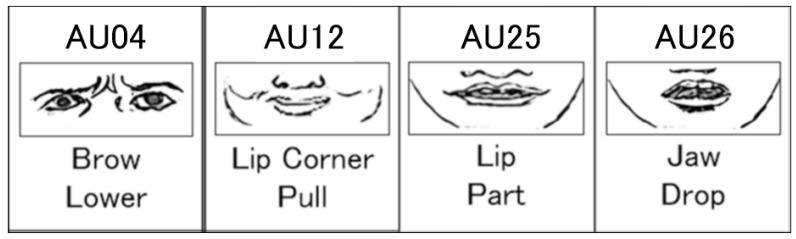
Facial Action Coding System action units (AUs) analyzed in the present study.

In a recent study [7], we presented participants with live performances of positive (smile) and negative (frown) dynamic facial expressions by the android Nikola (Figure 1 and Figure 2), which features 29 pneumatic actuators for facial actions and produces validated, human-like emotional facial expressions [5]. Participants’ corrugator supercilii and zygomaticus major EMG activity were recorded, and their faces were simultaneously videotaped. Nikola’s frowning expressions (negative condition) elicited stronger corrugator supercilii activity in the EMG data and increased brow-lowering (AU04, Figure 3) in the video recordings, whereas smiling expressions (positive condition) elicited stronger zygomaticus major activity and greater lip-corner puller activation (AU12, Figure 3). Both EMG and automated FACS analysis using Py-feat [22] previously confirmed participants’ spontaneous facial mimicry of the android’s dynamic facial expressions [7].

However, our previous study [7] had several limitations. First, the univariate peak-level statistics used did not account for the temporal dynamics of spontaneous facial mimicry. Second, facial mimicry involving AUs other than AU04 and AU12 was not evaluated. Third, the inference that Nikola induced participants’ spontaneous facial mimicry relied on the experimental design and prior knowledge that participants viewed Nikola’s dynamic facial expressions, whereas Nikola’s facial movements were determined solely by predefined actuator motions. Therefore, statistical evidence directly supporting a causal relationship between Nikola’s and participants’ facial expressions was lacking. Therefore, the objectives of the current study were: (1) to use multivariate time-series data and statistics to explore the nature of spontaneous facial mimicry beyond zygomaticus major/AU12 and corrugator supercilii/AU04 customarily measured in previous studies; (2) to determine the temporal latency of spontaneous facial mimicry elicited by the android Nikola; (3) to determine whether Granger causality could be assessed based on a temporal precedence effect of the mimickee (Nikola) on the mimickers (participants) during spontaneous facial mimicry; (4) to examine whether use of different statistical algorithms (participant muscular responses, time-series cross-correlations, and vector autoregression [VAR]) to evaluate the time-series data would yield converging results, e.g., whether the temporal precedence effect could be found at the lag with the maximal cross-correlation coefficient. For an overview of the theoretical and analytical frameworks of this study, please refer to Figure 1.

Univariate statistics, such as peak-level measures or cross-correlation analysis [17,23,24], are typically applied, as in the above-cited study, because EMG is usually recorded from only two or three muscles. Conversely, current automated FACS estimation from video data can simultaneously evaluate approximately 20 AUs, enabling the application of multivariate time-series statistics to detect behavioral matching [18,25,26]. However, most multivariate approaches are correlational in nature and do not provide evidence for causal inference. A notable exception is multivariate Granger causality, which is grounded in the principle of temporal precedence. In the context of multivariate Granger causality, given time series X, Y, and Z (with Z potentially comprising multiple time series), if the present X is predictive of the future of ***Y*** beyond the degree to which Y and Z already predict the future of Y, X is said to Granger cause Y [27,28,29]. Moreover, Granger causality has been applied in studies of interpersonal coordination [30,31,32]; however, it has not been applied specifically to facial mimicry. Several statistical frameworks are available to test Granger causality, most commonly VAR, which relies on three key assumptions: continuous-valued series, linear data-generating processes and causal effects, and discrete, regularly sampled data [33].

In the current study, we used Py-feat [22] to analyze video data from participants and the android. We derived trial-wise dyadic facial action time series. In each trial, the android’s face was presented to the participant for 3 s. We analyzed 105 frames per trial, corresponding to 3.5 s at 30 frames per second, to capture delayed responses in participants’ facial mimicry. Py-feat estimated AU time series are finite time series with continuous values and a regular sampling frequency, fulfilling two of the abovementioned assumptions about VAR—continuous-valued series of regularly sampled data. Multivariate VAR models can substantially increase the number of parameters to be estimated, depending on the numbers of time series and lags included.

To constrain the search for temporal effects to the most relevant AU time series, we conducted principal component analysis on Nikola’s 17 AU time series, with continuous values generated by Py-feat separately for the positive and negative conditions. We identified the two most contributory AU time series to Nikola’s smile and frown (AU25, lips part, and AU26, jaw drop, under both the positive and negative conditions; Figure 3), as those with the highest weights in the first principal component, in addition to AU12 for the positive condition and AU04 for the negative condition, as supported by past EMG-based studies. The rationale for selecting Nikola’s most contributory AUs was related to the definition of behavioral matching and mimicry. If Nikola activated an AU during a facial expression and the participant followed suit, we could be certain that the participant engaged in spontaneous facial mimicry. If Nikola did not activate an AU, and the participant also did not, then it would be unclear whether the participant “behaved” after Nikola’s non-expression, or if the participant simply remained static. We included three AUs per agent in the mlVAR models, because six time series is the maximum that could be estimated with correlated temporal random effects in the linear mixed effect model implementation in the multilevel VAR (*mlVAR*) package of R [34].

To investigate the mimicking temporal pattern of Nikola’s most contributing AUs, we performed cross-correlation analyses between the android and participant AU04, AU12, AU25, and AU26 time series, condition-wise, to test whether the cross-correlation coefficients exceeded zero. This identified time periods during which mimicry occurred.

To investigate the temporal patterns of android–human AU temporal precedence effects, we used the *mlVAR* package of R [34]. The mlVAR models both temporal and contemporaneous effects, as well as hierarchical between-subjects effects (Figure 1). The mlVAR model included three AUs from the dyadic data, yielding six AU time series. Although the experiment involved live performances by the android Nikola, the experimental design ensured that participants viewed Nikola’s dynamic facial expressions, whereas Nikola’s facial movements were determined solely by predefined actuator motions. Therefore, we expected to observe only the temporal effects of Nikola’s dynamic facial expressions on participants’ facial actions. We estimated all nine android–human AU temporal precedence effects across the time course to observe their dynamic evolution, and highlighted significant latencies to identify influential android–human effects. To the best of our knowledge, Granger causality has not yet been used to test spontaneous facial mimicry. We expected to observe dynamically changing temporal effects from android AUs to participant AUs. If android–human temporal effects occur across different AU pairs, it would indicate that facial responses when observing android facial expressions involve not only copying the motor pattern of individual AUs, but also the use of goal inferencing and motor planning during social cognition

This study excelled in the novel use of dyadic multivariate time-series data from both the android and participants, enabling estimation of interpretable Granger causality effects over time, and discussion in relation to the observed AU-paired cross-correlations. Both the approach and the findings are informative in terms of understanding the nature of spontaneous facial mimicry during human–android and human–human interactions [35,36]. There are also practical implications. The approach and findings can be applied to social androids to optimize performance in the commercial, financial, educational, and medical contexts.

## 2. Materials and Methods

### 2.1. Participants

Twenty-six adults (12 men and 14 women) were recruited in Kyoto, Japan (mean ± standard deviation [SD] of age = 25.27 ± 5.49 years, range: 20–37 years). They were all native Japanese with Japanese as their native language. The sample size was determined using an a priori power analysis conducted with G*Power software (ver. 3.1.9.2; Heinrich Heine University, Düsseldorf, Germany) [37]. The analysis was based on one-tailed paired *t*-tests to detect facial mimicry, specifically greater brow-lowering activity in response to angry expressions and greater lip-corner-pulling activity in response to happy expressions. The parameters included an α-level of 0.05 and a statistical power (1 − *β*) of 0.80, with an effect size (*d*) of 0.56 estimated from previous findings of visually detectable facial mimicry in response to human dynamic facial expressions [14]. This analysis indicated that a minimum of 22 participants was required. The study was approved by the Ethics Committee of RIKEN and conducted in accordance with its ethical standards. Written informed consent, including consent for video recording and publication, was obtained from all participants. All participants received financial compensation for their participation.

### 2.2. Facilities

The android Nikola [5] was used to present facial expressions, which was developed at RIKEN to study human–robot emotional interactions. Nikola’s face resembles a human child and is covered with soft, deformable silicone skin attached to the front of the skull. Beneath the skin, the android is equipped with 35 pneumatic actuators controlled by pressure valves: 29 actuators manage facial muscle movements, 3 control head movements, and 3 govern eyeball movements. A previous study [5] validated 17 of Nikola’s AUs associated with prototypical facial expressions, including AU04 (brow lowerer) and AU12 (lip-corner puller). The android receives motor control commands from a notebook computer (G-Tune P5-ADLABW11, Mouse Computer, Tokyo, Japan) running Ubuntu 20.04. Commands are transmitted via a USB cable, allowing Nikola to generate dynamic facial expressions. The android’s hair and make-up were kept consistent throughout the experiment.

To present Nikola’s facial expressions to participants and simultaneously videotape their faces, both the android and the participant faced a prompter (MPL-21W3/HDMI; Life-On, Hikone, Japan), consisting of a mirror reflecting a TV screen, with a video camera (VIXIA HF R800, Canon, Tokyo, Japan) concealed behind the mirror. Video signals were controlled by a switcher (SL-41C; Imagenics, Tokyo, Japan), which received serial port commands from a Windows 11 computer (Precision T3500, Dell, Round Rock, TX, USA) running Presentation 24.1 software (Neurobehavioral Systems, Berkeley, CA, USA) to execute the experimental paradigm and switch between the PC visual input and the android’s side camera. Video recordings were captured at a resolution of 1920 × 1080 pixels. Experiments were conducted individually in an electrically shielded soundproof room (Science Cabin; Takahashi Kensetsu, Tokyo, Japan).

### 2.3. Paradigm and Procedures

Upon arrival, participants were briefed on the study and provided informed consent before signing the consent form. An assistant prepared the participants and seated them in front of the prompter. During the initial phase of the experiment, participants engaged in passive viewing. They were instructed to focus on a central cross displayed on the screen, which appeared for a jittered duration of 2000–3750 ms, with an average intertrial interval of 2604 ms. During each trial, the android presented dynamic facial expressions of smiling (positive condition) and frowning (negative condition) for 3 s (Figure 2), including neutral, dynamic, and apex expressions each lasting 1 s. This timing has been shown to reflect natural changes in the dynamic facial expressions of human [38] and android [5] faces. Following each trial, the fixation cross reappeared on the screen. Participants completed four practice trials, comprising two trials for each emotion condition, and subsequently completed 30 passive viewing trials per condition, totaling 60 trials, with a scheduled break after 32 trials. Within each condition, 15 trials presented prototypical expressions based on AU descriptions of the happy and angry expressions in the FACS [5,20,39,40], and the rest presented Bayesian optimized expressions [41]. The latter were based on emotion-recognition ratings estimated by Py-Feat [22]. The resulting AU activation profile differed from that of the prototypical expressions. In addition, the optimized happy expression was rated as significantly less happy than the prototypical happy expression [41]. Therefore, only trials (15 per condition) presenting the prototypical expressions were analyzed. The order of conditions within the trials was pseudo-randomized for each participant to prevent more than three consecutive trials of the same condition (either positive or negative). Video data were recorded during these passive viewing trials. Afterward, participants provided 4 practice-ratings and 60 test-ratings of subjective experiential valence and arousal, during which no video data were acquired. These ratings were not analyzed in this study. Participants were remunerated for their participation and then dismissed.

### 2.4. Py-Feat Automated AU Estimation

The face recordings of participants and the android were segmented into 4 s clips corresponding to each trial, based on the timing recorded in the log files. All trials, including positive and negative conditions, underwent framewise automated FACS [20] estimation to extract AU amplitude time series. This was performed using Py-Feat [22] (version 0.6.0; Python 3.10) within a conda environment in Miniconda (version 23.10.0). The default modules were used for facial detection (RetinaFace), facial landmark estimation (MobileFaceNets), and AU estimation (XGBoost classifier). Py-Feat detects 20 AUs (01, 02, 04, 05, 06, 07, 09, 10, 12, 14, 15, 17, 18, 20, 23, 24, 25, 26, 28, and 43) as continuous values, with the exception of AUs 7, 11, and 20, which are classified as binary (1 or 0).

### 2.5. Statistical Analysis

#### 2.5.1. Principal Component Analysis

Apart from AU04 in the negative condition and AU12 in the positive condition, we performed principal component analysis using the *prcomp* function in R on the android’s 17 continuous AU time series to identify the AUs with the highest weights in the first principal component for each condition. In the negative condition, the first principal component explained 62.4% of the variance, with AU25 (lips part, weight = 0.634) and AU26 (jaw drop, weight = 0.433) contributing most. In the positive condition, the first principal component explained 64.6% of the variance, with AU25 (weight = 0.626) and AU26 (weight = 0.287) as the most heavily weighted. Therefore, AU25 and AU26 were identified as contributory AUs for both conditions.

#### 2.5.2. Cross-Correlations Between Android and Human AU Time Series

We cross-correlated trial-wise participant AU04, 12, 25, and 26 time series with the corresponding android time series up to 40 lags (at 30 fps, equivalent to 1333.333 ms) using the *crosscorr* function in MATLAB R2024b. Following a hierarchical approach, mean condition-wise cross-correlation series were computed and smoothed with a rolling window of five lags using the *rollmean* function in R version 4.5.2 for each participant. Group-level mean condition-wise cross-correlation series were estimated and visualized using base functions and packages *lsr* (0.5.2) and *ggplot2* (4.0.2) in R. The peak lag within each smoothed cross-correlation series was identified. We then performed one-sample *t*-tests using the *t.test* function in R on the cross-correlation coefficient series from the neurobiologically viable 5th to the 40th lags to assess the significance of the findings. Significant *t*-statistics identified time periods during which facial mimicry was present. The Benjamini–Hochberg procedure was used to derive the corrected false discovery rate (FDR). Additionally, peak cross-correlation coefficients were compared between the positive and negative conditions using paired *t*-tests. The Shapiro–Wilk normality test indicated that the peak AU12 cross-correlations under the positive condition were not normally distributed (*p* < 0.001). However, paired *t*-tests with more than 25 observations are robust to violations of the normality assumption [42]; therefore, both the standard *t*-test and Yuen’s robust *t*-test statistics are reported. Yuen’s robust paired *t*-test was performed using the *YuenTTest* function in the R package *DescTools* (0.99.60), with a trim level of 0.2 (the default, trimming 20% of the data), to compare peak cross-correlation coefficients between the positive and negative conditions. Such trimming resulted in a reduced degree of freedom (15 vs. 25) in Yuen’s *t*-test. Effect sizes were estimated using the Algina–Keselman–Penfield robust standardized difference (AKP), which is comparable to Cohen’s *d* [43], with calculations performed using the *dep.effect* function in the R package *WRS2* (1.1.7) [44]. All *t*-tests were one-tailed to reflect the directional nature of the hypotheses.

#### 2.5.3. mlVAR Estimation

MlVAR models were estimated using the *mlVAR* package (0.5.5) in R. For the negative condition, AU04, AU25, and AU26 from both the android and participants were included as variables to estimate temporal effects. For the positive condition, AU12, AU25, and AU26 from both the android and the participants were included to estimate temporal effects. Temporal random effects were assumed to be correlated. Three AUs per agent were included in the mlVAR models, because, in the *mlVAR* package, six time series in the model is the maximum that allows estimation of correlated temporal random effects in the linear mixed effect model implementation. Temporal effects were estimated up to the 40th lag. Each time lag was estimated separately in individual models to avoid multicollinearity among temporal effects across lags [45]. Although our experimental design primarily focused on the temporal effects of android AUs on participant AUs, the model estimated and controlled for the autoregressive and temporal effects of all six AUs (three from the participants and three from the android) included in the analysis. We report temporal effects from the 5th to the 40th lags, and also the corrected FDR obtained using the Benjamini–Hochberg procedure. To plot the beta value time courses of all nine android–participant AU temporal effects, we smoothed the beta time series with a rolling window of five lags using the *rollmean* function in R.

## 3. Results

### 3.1. Human-Android AU Cross-Correlations

#### 3.1.1. AU04 Cross-Correlation

**Figure 4:**
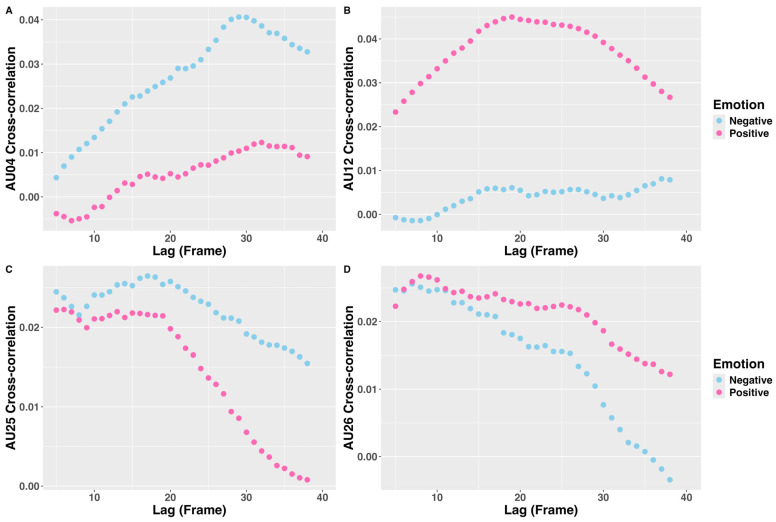
Group-average cross-correlations of android and participant action unit (AU) time series. Each *x*-axis represents the lag in frames (30 frames per second, 33 ms per frame) and each *y*-axis the cross-correlation coefficient. (**A**) Cross-correlation of dyadic AU04 time series: the peak occurred at the 29th lag under the negative condition and at the 32nd lag under the positive condition. (**B**) Cross-correlation of the dyadic AU12 time series: the peak occurred at the 19th lag under the positive condition and at the 37th lag under the negative condition. (**C**) Cross-correlation of dyadic AU25 time series: the peak occurred at the 6th lag under the positive condition and at the 17th lag under the negative condition. (**D**) Cross-correlation of dyadic AU26 time series: the peak occurred at the 8th lag under the positive condition and at the 7th lag under the negative condition.

Human–android AU04 cross-correlations reached peak values at different time lags depending on the condition. In the negative condition, the peak occurred at the 29th lag (966.667 ms). The group-level cross-correlation coefficients were significantly greater than zero from the 13th lag to the 40th lag, and all survived FDR correction. Under the positive condition, the peak appeared at the 32nd lag (1066.667 ms), and the cross-correlation coefficients were significantly larger than zero in lags 30, 33, and 34, but none survived FDR correction (Table 1). Comparison of group-level peaks indicated that the peak cross-correlation coefficient was significantly higher in the negative condition than in the positive condition (mean difference = 0.033, 95% CI = [0.012, Inf], *df* = 25, *t* = 2.673, *p* = 0.007, Cohen’s *d* = 0.524; Figure 4A).

#### 3.1.2. AU12 Cross-Correlations

In the positive condition, the human–android AU12 cross-correlation peak occurred at the 19th lag (633.333 ms), and the group-level cross-correlation coefficients were significantly greater than zero from the 14th lag to the 40th lag, all of which, apart from lags 14 and 15, survived FDR correction. Conversely, under the negative condition, the peak occurred at the 37th lag (1233.333 ms), but none of the cross-correlation coefficients was significantly different from zero (Table 2). Comparison of conditions showed that the standard *t*-test indicated a significantly higher peak cross-correlation coefficient in the positive condition than in the negative condition (mean difference = 0.038, 95% CI = [0.001, Inf], *df* = 25, *t* = 2.673, *p* = 0.048, Cohen’s *d* = 0.339), whereas the robust *t*-test did not yield a significant difference (trimmed mean difference = 0.014, 95% CI = [−0.018, 0.046], *df* = 15, *T_y_* = 0.938, *p* = 0.363, AKP = 0.123; Figure 4B).

#### 3.1.3. AU25 Cross-Correlations

The human–android AU25 cross-correlations attained peak values at different time lags depending on the condition. Under the positive condition, the peak appeared at the 6th lag (200 ms), and the group-level cross-correlation coefficients were significantly greater than zero from lag 5 to lag 21, except for lags 7 and 10. However, none survived the FDR correction. Under the negative condition, the peak occurred at the 17th lag (566.667 ms), and the group-level cross-correlation coefficients were significantly greater than zero from lag 5 to lag 40, except for lag 39, and all coefficients survived the FDR correction (Table 3). Comparison of group-level peaks indicated that the peak cross-correlation coefficient did not differ between the positive condition and the negative condition (mean difference = 0.004, 95% CI = [−0.020, Inf], *df* = 25, *t* = 0.311, *p* = 0.379, Cohen’s *d* = 0.061; Figure 4C).

#### 3.1.4. AU26 Cross-Correlations

The human–android AU26 cross-correlations attained peak values at different time lags depending on the condition. Under the positive condition, the peak appeared at the 8th lag (266.667 ms). The group-level cross-correlation coefficients were significantly greater than zero from lag 5 to lag 38, except for lag 37, and all coefficients survived the FDR correction. Under the negative condition, the peak occurred at the 7th lag (233.333 ms), and the group-level cross-correlation coefficients were significantly greater than zero from lag 5 to lag 27, except for lags 20, 23, 25, and 26. The coefficients survived the FDR correction, except for those of lags 16, 19, 21, 22, 24, and 27 (Table 4). Comparison of group-level peaks indicated that the peak cross-correlation coefficient was no different between the positive condition and the negative conditions (mean difference = 0.007, 95% CI = [−0.013, Inf], *df* = 25, *t* = 0.594, *p* = 0.279, Cohen’s *d* = 0.116; Figure 4D).

### 3.2. MlVAR Results

**Figure 5:**
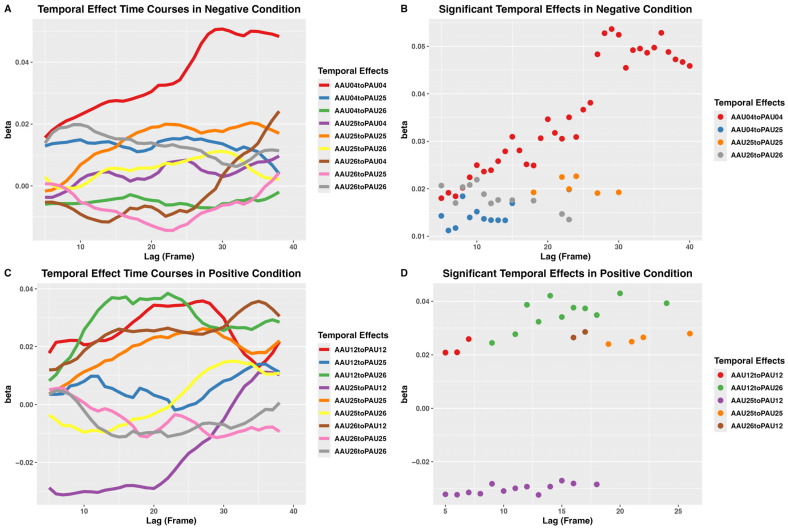
Action unit (AU) temporal precedence effects revealed by multilevel vector autoregression. Each *x*-axis represents the lag in frames (30 frames per second, 33 ms per frame) and each *y*-axis the beta coefficient of the directed temporal precedence effect. (**A**) Smoothed time course of temporal precedence effects under the negative condition. (**B**) Significant temporal precedence effects under the negative condition. (**C**) Smoothed time course of temporal precedence effects under the positive condition. (**D**) Significant temporal precedence effects under the positive condition. The time courses in (**A**,**C**) were smoothed with a rolling window of five lags using the *rollmean* function in R.

Under the negative condition, the temporal effects of android-to-participant AUs dynamically changed along the course of the trial (Figure 5A). The android AU04 amplitude significantly predicted the participant AU04 amplitude from the 5th lag to the 40th lag, and the temporal effects remained significant after FDR correction. The android AU04 amplitude also significantly predicted participant AU25 amplitude from the 5th to the 15th lags and at the 23rd lag. Lags 5 and 8 remained significant after FDR correction. The android AU25 amplitude significantly predicted participant AU25 amplitude at lags 18, 22, 23, 25, 27, and 30. However, these effects did not survive FDR correction. The android AU26 amplitude significantly predicted participant AU26 amplitude at lags 5, 7 to 13, 15, 18, 22, and 23, but none survived FDR correction (Figure 5B, Table 5).

Under the positive condition, android AU12 amplitude significantly predicted participant AU12 amplitude between the 6th and 8th lags. Android AU12 amplitude also significantly predicted participant AU26 amplitude at lags 9, 11–18, 20, and 24. Android AU25 amplitude significantly predicted participant AU25 amplitude at lags 19, 21, 22, and 26. Android AU25 amplitude also significantly predicted participant AU12 amplitude in a negative direction between the 5th and 16th lags and at the 18th lag. No temporal effects under the positive condition survived FDR correction (Figure 5C,D, Table 6).

## 4. Discussion

We examined the timing and temporal causal relationships in FACS data from simultaneous dyadic video recordings of participants viewing Nikola’s dynamic facial expressions in our previous study [7]. Under the negative condition, cross-correlation analysis indicated that mimicry of Nikola’s frowning expression in the brow lowerer (AU04) was significant from 433.333 ms onward. This finding corresponded with the mlVAR assessment of Granger causality, which showed that Nikola’s AU04 amplitudes exerted a significant temporal effect on participants’ AU04 amplitudes throughout the entire time-course. The mimicry of Nikola’s mouth-widening expression (AU25, lips part) was also significant throughout the entire time-course. The mlVAR revealed paired android–participant AU25 Granger causality between 600 and 1000 ms. The mimicry of Nikola’s jaw-dropping expression (AU26) was significant, between 166.667 and 900 ms, corresponding to the paired android–participant AU26 Granger causality between 166.667 and 766.667 ms.

Under the positive condition, for the lip-corner puller (AU12) mimicry of Nikola’s smiling expression, the cross-correlation was significant from 466.667 ms onwards. However, the mlVAR analysis revealed significant paired android–participant AU12 Granger causality between 166.667 and 233.333 ms. For mimicry of Nikola’s mouth-widening expression (AU25), the cross-correlation was significant, between 166.667 and 700 ms, whereas the paired android–participant AU25 Granger causality was observed between 633.333 and 866.667 ms. For mimicry of Nikola’s jaw-dropping expression (AU26), the cross-correlation was significant, between 166.667 and 1266.667 ms, but no paired android–participant AU26 Granger causality attained significance.

Turning the traditionally measured AU04/corrugator supercilii and AU12/zygomaticus major, cross-correlations indicated that human–android spontaneous facial mimicry exhibited a latency of 0.4–1 s, which falls within the range reported for human–human spontaneous facial mimicry of dynamic facial expressions (approximately 0.5–0.9 s) [14]. This finding is also consistent with the 1.18 s delay in human–android spontaneous facial mimicry reported by Hofree et al., 2014 [6], who used a different android, Einstein. The fact that observed spontaneous facial mimicry in human–android interactions has a latency comparable to that of human–human interactions implies that participants engage in behavioral matching with the android Nikola in a manner similar to that with a human counterpart. However, for the android–participant pairs of AU25 and AU26, the mimicry appeared to occur faster (from about 200 ms onwards) than for AU04 and AU12. To the best of our knowledge, previous studies of spontaneous facial mimicry have not investigated the timing of AU25 (depressor labii and orbicularis oris) and AU26 (masseter) mimicry responses. We thus lacked previous data for comparison. This is possibly attributable to earlier findings of high saliency in the mouth compared to other facial regions [46,47,48], which therefore attracted visual attention early and contributed to the rapid onset of participants’ responses in the mouth region. Future studies that incorporate simultaneous eye-tracking of participants’ visual attention are required to clarify this potential mechanism.

MlVAR revealed non-paired android–participant AU temporal precedence effects, in addition to paired android–participant AU Granger causality. Under the negative condition, android AU04 exerted temporal effects on participant AU25 between 166.667 and 766.667 ms. Under the positive condition, android AU12 exerted temporal effects on participant AU26 between 300 and 800 ms, whereas android AU25 exerted negative temporal effects on participant AU12 between 166.667 and 600 ms. This showed that the observed participant AU time series was influenced by multiple android AU actions, with temporal effects exerted at different times along the trial time course (Figure 5B,D), likely a consequence of both non-linear and temporal integrative effects. Therefore, the time course of the temporal precedence effects appeared different from that of the pairwise AU cross-correlations. Also, the time window of significant cross-correlation coefficients did not always correspond to the time window of significant Granger causality. Furthermore, the negative temporal effect from android AU25 to participant AU12 was unexpected but could be explained by an initial visual attentional focus on the salient mouth region, which may have suppressed or delayed the zygomaticus reaction relative to the mouth reaction, despite a concurrent early positive temporal effect of the android–participant AU12 pair.

The revealed android–human temporal effects across different AUs provide additional insight into the Goal Emulation, Planning, and Mimicry (EP-M) model [49]. According to this framework, if android–human temporal effects are observed only in identical AU pairs, spontaneous facial mimicry likely reflects a mechanical motor replica (the direct M route between the posterior temporal sulcus and inferior frontal gyrus). Conversely, if temporal effects occur across different AU pairs that are simultaneously active in the mimickee, mimicry may represent a more holistic response driven by top-down goal emulation, planning (the indirect emulation–planning [EP] route via the inferior parietal sulcus), or a mentalizing system-modulated social response [50]. Our mlVAR results support the possibility that the EP mechanism is involved in human–android interaction. In a previous neuroimaging study, Saygin et al. [51], demonstrated that, when observing the actions of the robotic form and the android form of android Repliee Q2, as well as the human on whom Repliee Q2 was based, using a repetition suppression paradigm, each participant’s mirror neuron system, including the inferior parietal sulcus, was more attuned with the action in the android form than the human and robotic forms. The cited authors, however, attributed the effect to the prediction error elicited by the android’s unnatural actions. However, spontaneous facial mimicry to the android does not necessarily require mentalistic attribution to androids. Given that the participants’ human likeness ratings did not correlate with the zygomaticus major/AU12 and corrugator supercilii/AU04 responses in the current dataset [7], our results cannot be used to imply the level of mentalistic attribution that the participants have towards the android, or to establish whether spontaneous facial mimicry was modulated by the participants’ level of mentalistic attribution towards the android [52]. However, the previously analyzed valence and arousal ratings of the current dataset showed that emotional contagion [2,4] was intact during human–android interactions with Nikola. It is important to investigate, in future studies, whether mentalistic attribution to androids modulates behavioral matching in human–android interactions.

It should be noted that other statistical implementations of Granger causality exist, including entropy transfer [29,53,54] and neural Granger causality [30,55], which can estimate non-linear temporal effects. However, the standard entropy transfer method is limited to bivariate data and relies on Shannon conditional entropy [56], which has issues such as positive bias and difficulty in assessing statistical significance [53]. Neural Granger causality approaches such as multilayer perceptrons or recurrent neural networks show promise for multivariate time-series data [30], provided that sufficient computational resources are available. Notably, despite the term “causality” [27], Granger causality estimates only temporal precedence, which is just one of several criteria needed to establish causality inference [57,58]. Therefore, interpretations of Granger causality should be made with caution.

Several limitations of this study should be acknowledged. First, we tested only happy (smiling) and angry (frowning) facial expressions. Hence, the effects of other expressions remain to be examined. Happy and angry expressions have been most often used to explore the effects of spontaneous facial mimicry [6,9,12,59], possibly because it is practical to detect (via surface EMG measurement) movements of the zygomaticus major and corrugator supercilii. The current study was originally designed to measure EMG, although we also collected dyadic facial video recordings, as also analyzed in the current study. However, the android Nikola shows valid facial expressions of all six basic emotions [5], as well as complex emotional states such as awe and “flirty face” [60]. The suggestion that Nikola is suitable for investigating a variety of emotions is a promising direction for future research. Second, the test setting was a laboratory, specifically a soundproof room, with both the android and the participant facing a prompter, and the android always against a sterile background. Although this setting ensured consistency across other visuo-audio conditions throughout the experiment, participants’ experiences of interacting with the android may have differed from those in more naturalistic social settings, such as in a commercial, financial, or medical context. Third, although the sample size of the current study was sufficient for drawing group inferences (see Section 2.1), it was too low to allow us to investigate the effects of individual differences [61]. Furthermore, for AU12 under the positive condition, previous analysis of zygomaticus major and AU12 responses via between-condition comparisons yielded significant results [7]. However, the peak cross-correlation coefficients were not normally distributed, and the between-condition comparison of the peak cross-correlation coefficients was significant only on the standard *t*-test, not when the robust Yuen’s trimmed mean test was applied. The non-normal distribution of the correlation coefficients, together with a larger standard error (Table 2), imply that individual variation was greater for AU12/zygomaticus mimicry than for other AU mimicries. Although paired *t*-tests with more than 25 observations are considered robust to violations of the normality assumption [42], a larger sample size should further help avoid such issues. It is important that future studies investigate how variables such as participants’ sex [62], age, socioeconomic status [63], trait empathy, autistic traits [64,65,66], social anxiety [67], and attitudes towards robots and technology [68,69] modulate spontaneous facial mimicry to robotic facial expressions.

## 5. Conclusions

By analyzing automated FACS-coded dyadic data in human–android interactions, we found that the latency of spontaneous facial mimicry in AU04 and AU12 was comparable to latencies reported in previous human–human interaction studies. We also found that AU25 and AU26 mimicry was more rapid than those of AU04 and AU12. MlVAR models revealed dynamically evolving Granger causality of paired android–human AUs, as well as temporal effects of different AU combinations, indicating that the spontaneous facial response when observing an android’s dynamic facial expressions does not involve only motor copying of facial movements, but also higher-level goal emulation and motor planning in the mirror mechanism. The multivariate, dynamic temporal effects could be further investigated, together with other concurrent behavioral or physiological measurements such as eye-tracking, to identify states and transitions during facial mimicry using a hidden Markov model. Future research should investigate individual differences that modulate human–android facial mimicry, such as sex [62], age, socioeconomic status [63], trait empathy, autistic traits [64,65,66], social anxiety [67], and attitudes towards robots and technology [68,69]. This approach may be valuable when detecting human–human or human–robot behavioral matching in real-life contexts, such as financial and commercial services, education, and medicine [70,71]. The latency and Granger causality findings for humans’ spontaneous facial mimicry in response to the dynamic facial expressions of the android Nikola further support the reliability of the social function of android facial expressions.

## Figures and Tables

**Table 1:** Significant action unit 04 cross-correlation coefficients.

Lag	Latency (ms)	Mean	*SE*	*t*	*p*	FDR	Cohen’s *d*
**Negative Condition**							
**13**	433.333	0.020	0.011	1.892	0.035	0.045	0.371
**14**	466.667	0.022	0.010	2.335	0.014	0.019	0.458
**15**	500.000	0.025	0.009	2.759	0.005	0.009	0.541
**16**	533.333	0.022	0.009	2.506	0.010	0.014	0.491
**17**	566.667	0.023	0.009	2.564	0.008	0.013	0.503
**18**	600.000	0.021	0.009	2.346	0.014	0.019	0.460
**19**	633.333	0.028	0.009	3.090	0.002	0.004	0.606
**20**	666.667	0.031	0.009	3.263	0.002	0.003	0.640
**21**	700.000	0.027	0.010	2.724	0.006	0.009	0.534
**22**	733.333	0.028	0.008	3.452	0.001	0.002	0.677
**23**	766.667	0.032	0.008	3.805	0.000	0.001	0.746
**24**	800.000	0.028	0.008	3.265	0.002	0.003	0.640
**25**	833.333	0.033	0.008	4.048	0.000	0.001	0.794
**26**	866.667	0.034	0.008	4.071	0.000	0.001	0.798
**27**	900.000	0.039	0.009	4.607	0.000	0.001	0.904
**28**	933.333	0.042	0.009	4.958	0.000	0.001	0.972
**29**	966.667	0.043	0.009	4.570	0.000	0.001	0.896
**30**	1000.000	0.042	0.009	4.682	0.000	0.001	0.918
**31**	1033.333	0.037	0.009	4.021	0.000	0.001	0.789
**32**	1066.667	0.039	0.010	4.052	0.000	0.001	0.795
**33**	1100.000	0.038	0.009	4.076	0.000	0.001	0.799
**34**	1133.333	0.037	0.009	3.979	0.000	0.001	0.780
**35**	1166.667	0.035	0.010	3.452	0.001	0.002	0.677
**36**	1200.000	0.036	0.010	3.685	0.001	0.002	0.723
**37**	1233.333	0.033	0.009	3.573	0.001	0.002	0.701
**38**	1266.667	0.031	0.010	3.228	0.002	0.003	0.633
**39**	1300.000	0.033	0.009	3.701	0.001	0.002	0.726
**40**	1333.333	0.031	0.009	3.461	0.001	0.002	0.679
**Positive Condition**							
**30**	1000.000	0.014	0.008	1.790	0.043	0.418	0.351
**33**	1100.000	0.013	0.007	1.771	0.044	0.418	0.347
**34**	1133.333	0.016	0.007	2.246	0.017	0.418	0.440

Abbreviations. FDR: false discovery rate (Benjamini–Hochberg procedure); *p*: *p*-values; *SE*: standard error; *t*: *t*-statistics.

**Table 2:** Significant action unit 12 cross-correlation coefficients under the positive condition.

Lag	Latency (ms)	Mean	*SE*	*t*	*p*	FDR	Cohen’s *d*
**14**	466.667	0.041	0.023	1.809	0.041	0.057	0.355
**15**	500.000	0.039	0.022	1.770	0.044	0.059	0.347
**16**	533.333	0.043	0.022	1.958	0.031	0.044	0.384
**17**	566.667	0.048	0.022	2.187	0.019	0.034	0.429
**18**	600.000	0.045	0.022	2.052	0.025	0.040	0.402
**19**	633.333	0.045	0.021	2.115	0.022	0.036	0.415
**20**	666.667	0.043	0.021	2.020	0.027	0.041	0.396
**21**	700.000	0.044	0.021	2.159	0.020	0.035	0.423
**22**	733.333	0.045	0.020	2.282	0.016	0.031	0.448
**23**	766.667	0.044	0.019	2.242	0.017	0.032	0.440
**24**	800.000	0.043	0.019	2.325	0.014	0.030	0.456
**25**	833.333	0.043	0.018	2.375	0.013	0.029	0.466
**26**	866.667	0.042	0.018	2.374	0.013	0.029	0.466
**27**	900.000	0.044	0.017	2.620	0.007	0.020	0.514
**28**	933.333	0.042	0.016	2.601	0.008	0.020	0.510
**29**	966.667	0.041	0.015	2.652	0.007	0.020	0.520
**30**	1000.000	0.038	0.014	2.672	0.007	0.020	0.524
**31**	1033.333	0.037	0.014	2.750	0.005	0.020	0.539
**32**	1066.667	0.037	0.013	2.915	0.004	0.020	0.572
**33**	1100.000	0.035	0.012	2.946	0.003	0.020	0.578
**34**	1133.333	0.033	0.011	2.946	0.003	0.020	0.578
**35**	1166.667	0.032	0.011	3.003	0.003	0.020	0.589
**36**	1200.000	0.029	0.011	2.698	0.006	0.020	0.529
**37**	1233.333	0.027	0.010	2.637	0.007	0.020	0.517
**38**	1266.667	0.027	0.010	2.852	0.004	0.020	0.559
**39**	1300.000	0.025	0.009	2.685	0.006	0.020	0.527
**40**	1333.333	0.025	0.009	2.845	0.004	0.020	0.558

Abbreviations. FDR: false discovery rate (Benjamini–Hochberg procedure); *p*: *p*-values; *SE*: standard error; *t*: *t*-statistics.

**Table 3:** Significant action unit 25 cross-correlation coefficients.

Lag	Latency (ms)	Mean	*SE*	*t*	*p*	FDR	Cohen’s *d*
**Positive Condition**							
**5**	166.667	0.023	0.010	2.265	0.016	0.099	0.444
**6**	200.000	0.029	0.011	2.535	0.009	0.099	0.497
**8**	266.667	0.020	0.011	1.813	0.041	0.099	0.355
**9**	300.000	0.020	0.010	1.879	0.036	0.099	0.368
**11**	366.667	0.024	0.010	2.439	0.011	0.099	0.478
**12**	400.000	0.024	0.010	2.349	0.014	0.099	0.461
**13**	433.333	0.020	0.011	1.896	0.035	0.099	0.372
**14**	466.667	0.022	0.011	1.970	0.030	0.099	0.386
**15**	500.000	0.021	0.010	2.128	0.022	0.099	0.417
**16**	533.333	0.020	0.011	1.887	0.035	0.099	0.370
**17**	566.667	0.026	0.010	2.636	0.007	0.099	0.517
**18**	600.000	0.020	0.011	1.833	0.039	0.099	0.359
**19**	633.333	0.021	0.011	1.810	0.041	0.099	0.355
**20**	666.667	0.020	0.011	1.897	0.035	0.099	0.372
**21**	700.000	0.020	0.010	1.982	0.029	0.099	0.389
**Negative Condition**							
**5**	166.667	0.027	0.009	3.007	0.003	0.012	0.590
**6**	200.000	0.022	0.008	2.587	0.008	0.014	0.507
**7**	233.333	0.019	0.010	1.941	0.032	0.033	0.381
**8**	266.667	0.024	0.009	2.753	0.005	0.012	0.540
**9**	300.000	0.022	0.009	2.498	0.010	0.015	0.490
**10**	333.333	0.021	0.009	2.464	0.010	0.016	0.483
**11**	366.667	0.027	0.009	3.026	0.003	0.012	0.593
**12**	400.000	0.026	0.008	3.048	0.003	0.012	0.598
**13**	433.333	0.024	0.009	2.718	0.006	0.012	0.533
**14**	466.667	0.025	0.009	2.797	0.005	0.012	0.549
**15**	500.000	0.026	0.009	2.719	0.006	0.012	0.533
**16**	533.333	0.028	0.010	2.931	0.004	0.012	0.575
**17**	566.667	0.024	0.009	2.573	0.008	0.014	0.505
**18**	600.000	0.028	0.008	3.389	0.001	0.012	0.665
**19**	633.333	0.026	0.009	2.872	0.004	0.012	0.563
**20**	666.667	0.025	0.009	2.769	0.005	0.012	0.543
**21**	700.000	0.024	0.009	2.762	0.005	0.012	0.542
**22**	733.333	0.026	0.009	2.788	0.005	0.012	0.547
**23**	766.667	0.025	0.009	2.755	0.005	0.012	0.540
**24**	800.000	0.023	0.008	2.750	0.005	0.012	0.539
**25**	833.333	0.021	0.009	2.318	0.014	0.019	0.455
**26**	866.667	0.021	0.008	2.740	0.006	0.012	0.537
**27**	900.000	0.024	0.009	2.790	0.005	0.012	0.547
**28**	933.333	0.020	0.008	2.564	0.008	0.014	0.503
**29**	966.667	0.020	0.008	2.364	0.013	0.018	0.464
**30**	1000.000	0.021	0.008	2.580	0.008	0.014	0.506
**31**	1033.333	0.019	0.008	2.379	0.013	0.018	0.467
**32**	1066.667	0.016	0.008	1.947	0.031	0.033	0.382
**33**	1100.000	0.018	0.008	2.132	0.022	0.027	0.418
**34**	1133.333	0.017	0.008	2.002	0.028	0.032	0.393
**35**	1166.667	0.019	0.008	2.334	0.014	0.019	0.458
**36**	1200.000	0.019	0.008	2.533	0.009	0.015	0.497
**37**	1233.333	0.015	0.007	1.959	0.031	0.033	0.384
**38**	1266.667	0.016	0.008	2.011	0.028	0.032	0.394
**40**	1333.333	0.015	0.007	2.003	0.028	0.032	0.393

Abbreviations. FDR: false discovery rate (Benjamini–Hochberg procedure); *p*: *p*-values; *SE*: standard error; *t*: *t*-statistics.

**Table 4:** Significant action unit 26 cross-correlation coefficients.

Lag	Latency (ms)	Mean	*SE*	*t*	*p*	FDR	Cohen’s *d*
**Positive Condition**							
**5**	166.667	0.020	0.009	2.197	0.019	0.040	0.431
**6**	200.000	0.028	0.009	3.131	0.002	0.040	0.614
**7**	233.333	0.025	0.010	2.615	0.007	0.040	0.513
**8**	266.667	0.030	0.010	3.089	0.002	0.040	0.606
**9**	300.000	0.026	0.010	2.492	0.010	0.040	0.489
**10**	333.333	0.025	0.009	2.602	0.008	0.040	0.510
**11**	366.667	0.027	0.010	2.751	0.005	0.040	0.539
**12**	400.000	0.023	0.010	2.308	0.015	0.040	0.453
**13**	433.333	0.024	0.010	2.343	0.014	0.040	0.459
**14**	466.667	0.023	0.011	2.148	0.021	0.040	0.421
**15**	500.000	0.026	0.011	2.333	0.014	0.040	0.458
**16**	533.333	0.023	0.010	2.288	0.015	0.040	0.449
**17**	566.667	0.022	0.011	1.965	0.030	0.042	0.385
**18**	600.000	0.025	0.012	2.135	0.021	0.040	0.419
**19**	633.333	0.025	0.011	2.222	0.018	0.040	0.436
**20**	666.667	0.021	0.012	1.860	0.037	0.046	0.365
**21**	700.000	0.022	0.012	1.787	0.043	0.048	0.351
**22**	733.333	0.020	0.011	1.760	0.045	0.049	0.345
**23**	766.667	0.025	0.012	2.161	0.020	0.040	0.424
**24**	800.000	0.021	0.012	1.861	0.037	0.046	0.365
**25**	833.333	0.022	0.011	2.016	0.027	0.042	0.395
**26**	866.667	0.023	0.010	2.213	0.018	0.040	0.434
**27**	900.000	0.021	0.010	2.024	0.027	0.042	0.397
**28**	933.333	0.024	0.009	2.608	0.008	0.040	0.511
**29**	966.667	0.019	0.009	2.170	0.020	0.040	0.426
**30**	1000.000	0.018	0.008	2.095	0.023	0.041	0.411
**31**	1033.333	0.017	0.009	1.929	0.033	0.043	0.378
**32**	1066.667	0.015	0.008	1.998	0.028	0.042	0.392
**33**	1100.000	0.014	0.007	1.827	0.040	0.046	0.358
**34**	1133.333	0.016	0.007	2.215	0.018	0.040	0.434
**35**	1166.667	0.014	0.007	2.084	0.024	0.041	0.409
**36**	1200.000	0.013	0.007	1.981	0.029	0.042	0.389
**38**	1266.667	0.013	0.007	1.826	0.040	0.046	0.358
**Negative Condition**							
**5**	166.667	0.028	0.010	2.873	0.004	0.041	0.563
**6**	200.000	0.026	0.010	2.728	0.006	0.041	0.535
**7**	233.333	0.023	0.010	2.341	0.014	0.041	0.459
**8**	266.667	0.023	0.009	2.389	0.012	0.041	0.468
**9**	300.000	0.028	0.009	3.025	0.003	0.041	0.593
**10**	333.333	0.025	0.009	2.629	0.007	0.041	0.516
**11**	366.667	0.023	0.010	2.396	0.012	0.041	0.470
**12**	400.000	0.024	0.010	2.442	0.011	0.041	0.479
**13**	433.333	0.022	0.009	2.394	0.012	0.041	0.469
**14**	466.667	0.019	0.009	2.112	0.022	0.058	0.414
**15**	500.000	0.025	0.010	2.632	0.007	0.041	0.516
**16**	533.333	0.019	0.010	1.980	0.029	0.062	0.388
**17**	566.667	0.020	0.009	2.377	0.013	0.041	0.466
**18**	600.000	0.021	0.009	2.512	0.009	0.041	0.493
**19**	633.333	0.018	0.008	2.200	0.019	0.052	0.431
**21**	700.000	0.018	0.009	1.853	0.038	0.072	0.363
**22**	733.333	0.018	0.009	1.993	0.029	0.062	0.391
**24**	800.000	0.018	0.009	1.989	0.029	0.062	0.390
**27**	900.000	0.017	0.009	1.953	0.031	0.062	0.383

Abbreviations. FDR: false discovery rate (Benjamini–Hochberg procedure); *p*: *p*-values; *SE*: standard error; *t*: *t*-statistics.

**Table 5:** Significant android–human action unit (AU) temporal effects under the negative condition.

Lag (Frame)	Latency (ms)	*β*	*SE*	*p*	FDR
**Android AU04 to Participant AU04**					
**5**	166.667	0.018	0.008	0.028	0.029
**6**	200.000	0.019	0.008	0.018	0.020
**7**	233.333	0.018	0.009	0.032	0.032
**8**	266.667	0.020	0.009	0.020	0.021
**9**	300.000	0.022	0.010	0.020	0.021
**10**	333.333	0.025	0.010	0.010	0.013
**11**	366.667	0.024	0.010	0.014	0.018
**12**	400.000	0.024	0.010	0.016	0.019
**13**	433.333	0.026	0.011	0.017	0.020
**14**	466.667	0.028	0.010	0.004	0.006
**15**	500.000	0.031	0.009	0.000	0.004
**16**	533.333	0.028	0.010	0.006	0.009
**17**	566.667	0.025	0.010	0.014	0.018
**18**	600.000	0.025	0.009	0.007	0.009
**19**	633.333	0.031	0.010	0.002	0.005
**20**	666.667	0.035	0.011	0.001	0.005
**21**	700.000	0.032	0.011	0.003	0.006
**22**	733.333	0.031	0.011	0.005	0.008
**23**	766.667	0.035	0.011	0.002	0.005
**24**	800.000	0.031	0.011	0.005	0.008
**25**	833.333	0.037	0.012	0.002	0.005
**26**	866.667	0.038	0.012	0.002	0.005
**27**	900.000	0.048	0.013	0.000	0.003
**28**	933.333	0.053	0.014	0.000	0.002
**29**	966.667	0.054	0.014	0.000	0.002
**30**	1000.000	0.052	0.014	0.000	0.002
**31**	1033.333	0.045	0.014	0.001	0.005
**32**	1066.667	0.049	0.015	0.001	0.005
**33**	1100.000	0.050	0.015	0.001	0.005
**34**	1133.333	0.049	0.015	0.002	0.005
**35**	1166.667	0.050	0.016	0.002	0.005
**36**	1200.000	0.053	0.016	0.001	0.005
**37**	1233.333	0.049	0.015	0.002	0.005
**38**	1266.667	0.047	0.016	0.004	0.006
**39**	1300.000	0.047	0.015	0.002	0.005
**40**	1333.333	0.046	0.017	0.006	0.009
**Android AU04 to Participant AU25**					
**5**	166.667	0.014	0.005	0.003	0.046
**6**	200.000	0.011	0.005	0.025	0.101
**7**	233.333	0.012	0.005	0.017	0.078
**8**	266.667	0.018	0.004	0.000	0.000
**9**	300.000	0.014	0.005	0.011	0.060
**10**	333.333	0.015	0.005	0.005	0.060
**11**	366.667	0.014	0.005	0.012	0.060
**12**	400.000	0.013	0.005	0.007	0.060
**13**	433.333	0.013	0.006	0.039	0.130
**14**	466.667	0.013	0.007	0.043	0.130
**15**	500.000	0.017	0.007	0.011	0.060
**23**	766.667	0.020	0.010	0.041	0.130
**Android AU25 to Participant AU25**					
**18**	600.000	0.019	0.009	0.039	0.164
**22**	733.333	0.022	0.010	0.019	0.164
**23**	766.667	0.020	0.010	0.038	0.164
**24**	800.000	0.023	0.010	0.020	0.164
**27**	900.000	0.019	0.008	0.016	0.164
**30**	1000.000	0.019	0.009	0.036	0.164
**Android AU26 to Participant AU26**					
**5**	166.667	0.021	0.009	0.019	0.145
**7**	233.333	0.017	0.008	0.033	0.145
**8**	266.667	0.020	0.008	0.013	0.145
**9**	300.000	0.021	0.010	0.029	0.145
**10**	333.333	0.022	0.009	0.018	0.145
**11**	366.667	0.019	0.009	0.036	0.145
**12**	400.000	0.017	0.009	0.049	0.146
**13**	433.333	0.018	0.008	0.035	0.145
**15**	500.000	0.018	0.008	0.033	0.145
**18**	600.000	0.017	0.008	0.031	0.145
**22**	733.333	0.015	0.007	0.043	0.146
**23**	766.667	0.014	0.007	0.048	0.146

Abbreviations. *β*: beta coefficient; FDR: false discovery rate (Benjamini–Hochberg procedure); *p*: *p*-values; *SE*: standard error.

**Table 6:** Significant android–human action unit (AU) temporal effects under the positive condition.

Lag (Frame)	Latency (ms)	*β*	*SE*	*p*	FDR
**Android AU12 to Participant AU12**					
**5**	166.667	0.021	0.047	0.047	0.265
**6**	200.000	0.021	0.044	0.044	0.265
**7**	233.333	0.026	0.030	0.030	0.265
**Android AU12 to Participant AU26**					
**9**	300.000	0.024	0.045	0.045	0.136
**11**	366.667	0.028	0.032	0.032	0.127
**12**	400.000	0.039	0.003	0.003	0.083
**13**	433.333	0.032	0.017	0.017	0.085
**14**	466.667	0.042	0.005	0.005	0.083
**15**	500.000	0.034	0.014	0.014	0.083
**16**	533.333	0.038	0.013	0.013	0.083
**17**	566.667	0.037	0.012	0.012	0.083
**18**	600.000	0.035	0.020	0.020	0.089
**20**	666.667	0.043	0.010	0.010	0.083
**24**	800.000	0.039	0.035	0.035	0.127
**Android AU25 to Participant AU25**					
**19**	633.333	0.024	0.040	0.040	0.227
**21**	700.000	0.025	0.038	0.038	0.227
**22**	733.333	0.026	0.016	0.016	0.227
**26**	866.667	0.028	0.042	0.042	0.227
**Android AU25 to Participant AU12**					
**5**	166.667	−0.032	0.018	0.018	0.108
**6**	200.000	−0.032	0.019	0.019	0.108
**7**	233.333	−0.032	0.018	0.018	0.108
**8**	266.667	−0.032	0.014	0.014	0.108
**9**	300.000	−0.028	0.027	0.027	0.108
**10**	333.333	−0.031	0.008	0.008	0.108
**11**	366.667	−0.030	0.027	0.027	0.108
**12**	400.000	−0.029	0.029	0.029	0.108
**13**	433.333	−0.032	0.017	0.017	0.108
**14**	466.667	−0.029	0.030	0.030	0.108
**15**	500.000	−0.027	0.035	0.035	0.108
**16**	533.333	−0.028	0.036	0.036	0.108
**18**	600.000	−0.028	0.049	0.049	0.135

Abbreviations. *β*: beta coefficient; FDR: false discovery rate (Benjamini–Hochberg procedure); *p*: *p*-values; *SE*: standard error.

## Data Availability

The data and scripts presented in this study are openly available on the depository of the Open Science Framework at https://doi.org/10.17605/OSF.IO/MBDCA (DOI created and accessed on 6 February 2026).

## Abbreviations

The following abbreviations are used in this manuscript:

AU	Action unit
EMG	Electromyography
FACS	Facial Action Coding System
FDR	False discovery rate
mlVAR	Multilevel vector autoregression
SE	Standard error
